# Italian Systemic Lupus Erythematosus (SLE) Patients: Overview of Their Quality of Life and Unmet Needs

**DOI:** 10.3390/jcm14238498

**Published:** 2025-11-30

**Authors:** Luca Moroni, Ginevra De Marchi, Rosa Pelissero, Mercedes Callori, Italia Agresta, Antonella Celano, Elisa Cosentino, Silvia Tamanini, Alessia Delli Carri, Giuseppe Alvise Ramirez, Alessia Nano, Luca Quartuccio, Lorenzo Dagna

**Affiliations:** 1 Unit of Immunology, Rheumatology, Allergy and Rare Disease (UniRAR), IRCCS San Raffaele Hospital, 20132 Milan, Italy; 2 Faculty of Medicine, Vita-Salute San Raffaele University, 20132 Milan, Italy; 3 Division of Rheumatology, Azienda Sanitaria Universitaria Friuli Centrale (ASUFC), 33100 Udine, Italy; 4 Gruppo LES Italiano ODV, 10040 Leini, Italy; 5 APMARR Associazione Nazionale Persone con Malattie Reumatologiche e Rare APS ETS, 73100 Lecce, Italy; 6 Medical Affairs, GlaxoSmithKline S.p.A., 37135 Verona, Italy

**Keywords:** systemic lupus erythematosus, advisory board, unmet needs, quality of life, Italian patients with SLE

## Abstract

**Background/Objectives**: Systemic Lupus Erythematosus (SLE) is a multifactorial disease that significantly affects patients’ quality of life (QoL) and poses management challenges. This project combined a nationwide patient listening initiative with an Advisory Board (AB) to identify unmet needs and perceptions, aiming to integrate patient perspectives into decision-making and enhance SLE care. **Methods**: The “PaLESiamoci!” project, conducted by IQVIA with two patient organizations (Gruppo LES Italiano and APMARR), included both qualitative and quantitative phases. Ten patients completed disease diaries and one-hour interviews, while 151 others voluntarily filled a PO-administered survey including the validated 12-item Medical Outcome Short Form (SF-12) and items on physical, emotional, and daily life impact. Insights from these phases informed the AB discussion involving clinicians and patient representatives on six key topics. **Results**: Patients with SLE showed lower Physical (−13.4) and Mental (−14.0) Component Summary scores than the Italian population, indicating reduced social and work functioning. Despite EULAR recommendations to minimize corticosteroid use, 64% of patients remained on corticosteroids. The AB discussions revealed key unmet needs, including differing awareness of organ damage risks, corticosteroid-related adverse events, and adherence challenges, as well as the need for non-pharmacological and multidisciplinary support. **Conclusions:** The project highlighted major gaps and opportunities in SLE management. Patient organizations and rheumatologists emphasized developing tailored educational materials, strengthening rheumatologist–patient communication, and promoting multidisciplinary, patient-centered approaches to improve overall care.

## 1. Introduction

Systemic Lupus Erythematosus (SLE) is a complex autoimmune disease characterized by a broad spectrum of multi-systemic manifestations. Epidemiological data show that SLE has a disproportionately varied incidence and prevalence worldwide, with reported rates ranging from 1.5 to 11.0 per 100,000 person-years and from 13.0 to 7713.5 per 100,000 individuals, respectively [1]. An Italian observational study analyzing data from 2017 to 2022 estimated a mean incidence rate of 6.51 cases per 100,000 person-years, and mean prevalence of 60.57 per 100,000 people [2]. Variations in epidemiological estimates may also result from differences in the application of classification criteria, diversity in healthcare systems [3].

Challenges in the diagnostic process further burden patients, significantly impacting their quality of life (QoL). According to The European Alliance of Associations for Rheumatology (EULAR), a comprehensive evaluation of patients with SLE must include assessments of QoL, alongside disease progression and related damages [4]. Studies have demonstrated a correlation between irreversible organ damage in patients with SLE and their diminished QoL, emphasizing the need to consider both clinical and psychosocial factors when evaluating patient well-being [5,6].

Few studies highlighted the importance of assessing QoL in patients with SLE using patient-reported outcome measures (PROMs) particularly the 12-item Short Form (SF-12), which has proven effective in this context [7,8,9,10,11]. In light of this, there is increasing recognition of the value of integrating patient experiences into healthcare planning and research to better align services with real-world needs.

The initiative was designed to explore the experiences of individuals living with SLE throughout the entire care continuum. This project amplified patient voices through structured diaries, surveys, and advisory board (AB) discussions, providing both qualitative and quantitative insights into disease management, healthcare accessibility, and the broader impact of SLE on daily life of patients.

Characterizing the experiences of Italian patients with SLE and understanding the factors affecting their QoL, critical challenges, and unmet needs may facilitate the development of strategies to improve patient care. Insights from this project can help identify patient-defined priorities to inform research, clinical practice, and health policy.

## 2. Materials and Methods

**Figure 1:**
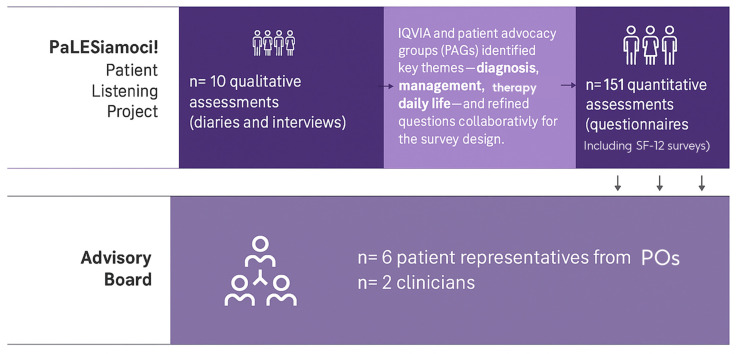
Flow Chart.

“PaLESiamoci!” was an in-depth patient listening project led by IQVIA (Survey service provider) in collaboration with two patient organizations (POs): Gruppo LES Italiano ODV and the Associazione Nazionale Persone con Malattie Reumatologiche e Rare—APMARR. The project utilized a mixed-methods design, combining both qualitative and quantitative analyses to explore the QoL and unmet needs of Italian patients with SLE. The qualitative analysis aimed to gain an in-depth understanding of the lived experiences, unmet needs, and therapeutic perception of patients with SLE in Italy serving as an exploratory investigation. Then, this was followed by a quantitative analysis that included the patient listening phase and culminated in an AB, which engaged both patient representatives and clinicians. The AB was performed in 2024 (Figure 1).

This research was non-clinical and non-interventional, intended solely for patient insight purposes. The project was designed as a patient-led initiative in collaboration with POs, with a primary focus on lived experiences rather than physician-assessed measures of disease activity or organ damage. Standard clinical indices were not included, as the goal was to explore the patient’s perspective on QoL and daily challenges.

### 2.1. Qualitative Analysis

Initially IQVIA and POs jointly defined the key areas to explore during the qualitative phase. Subsequently, ten voluntary patients identified by the POs completed narrative diaries (Appendix A.1) over a one-week period, followed by 1 h remote interviews (Appendix A.1). The diary structure and interview guide were developed by IQVIA and shared with the POs prior to data collection.

Patient recruitment was agreed upon between IQVIA and the POs, carried out through the Gruppo LES Italiano and APMARR, with the following target characteristics: patients with SLE for several years, with organ involvement, no overlap with other conditions, mixed age and gender, not too knowledgeable about SLE.

IQVIA contacted the selected patients, collected their diaries, and conducted the interviews.

This initial phase aimed to collect in-depth insights into patient journey, covering diagnosis, patient management, follow-up procedures, therapy experiences, and the daily challenges of living with SLE. An inductive analysis of the qualitative data was conducted by IQVIA to identify key themes, which were then used to inform and shape the design of the quantitative questionnaire.

### 2.2. Quantitative Analysis

In the quantitative phase, questionnaires (Appendix A.2) were voluntarily completed by the patients recruited through the communication channels of the POs over a one-month period. A purposive sampling strategy was applied, and the survey link was distributed by POs via social media channels. It ensured full anonymity and prevented the traceability of any personal data by either IQVIA or the POs. Patients were already familiar with questionnaires, as these are commonly used by POs. In this case, the survey was shared through their usual communication channels with a brief introduction explaining the purpose of the “PaLESiamoci!” project.

The questionnaire was designed to be self-administered and easily understandable, requiring no additional training. A cohort of 151 patients participated in a comprehensive patient journey survey using a Computer-Assisted Web Interviewing (CAWI) methodology. The survey consisted of both closed- and open-ended questions exploring diagnostic experience, QoL, treatment perceptions, and care pathways. It incorporated the literature-validated 12-item Medical Outcome Short Form (SF-12) [10] and a series of questions addressing physical, emotional, and daily life perception. SF-12 results were compared with data from the general Italian population, as reported in the Istat BES Report 2014 [12].

In the second part of the survey, patients responded to targeted questions regarding symptom prevalence and perceptions, the impact of SLE on daily life, employment, leisure activities, the diagnostic process, follow-up care, therapeutic regimen, critical challenges, and unmet needs.

The entire survey took approximately 20 min per participant.

To account for variability in disease burden, patients self-reported their perceived SLE severity. Based on their responses, participants were stratified into three categories, mild, moderate, and severe SLE, allowing for subgroup analyses to explore how disease severity influenced QoL, symptom burden, and treatment adherence.

Quantitative data were analyzed using descriptive statistics via SAS software (v9.4).

The sample, though relatively small, reflects the gender, age and regional distribution observed in national epidemiological studies on SLE. This size aligns with other exploratory, qualitative research studies in niche patient populations.

For qualitative data, an inductive thematic approach was employed which allowed key themes to emerge directly from the data. Key findings related to adherence and patients’ awareness of disease progression risk were used to inform the subsequent AB discussions.

### 2.3. Advisory Board Meeting

Following the quantitative analysis, a multidisciplinary AB was convened with two rheumatologists and six representatives from POs, to collect and interpret advice on topics raised during the patient listening phase. The objective was to explore actionable insights, particularly around treatment adherence and patients’ awareness of disease progression, emerging from the quantitative data. Appendix A.3 outlines the six topics. The AB was structured into three moderated sessions:

- The six patient representatives discussed the topics independently.
- Separately, the two rheumatologists discussed the same topics.
- Afterwards, a joint session allowed patients and clinicians to exchange views, resolve discrepancies, and build consensus.

Each session was moderated by experienced facilitators, and the discussions were anonymized and transcribed. The feedback gathered was used to contextualize findings and identify areas for clinical and policy-level improvement. Following the meeting, a comprehensive report summarizing the final insights and conclusions was produced and subsequently shared with all participants.

### 2.4. Patient and Public Involvement Statement (PPI)

POs were actively involved at multiple stages of this project, including data collection, and interpretation of findings.

Two national Italian POs, Gruppo LES Italiano (focused on Patients with SLE) and Associazione Nazionale Persone con Malattie Reumatologiche e Rare—APMARR (dedicated to patients with rheumatological diseases including SLE), collaborated as key partners in the project “PaLESiamoci!” conducted by IQVIA, providing input on project priorities and supporting recruitment. PO representatives played a crucial role in shaping the focus of the project, ensuring that the research questions and survey instruments addressed real-world concerns. The AB, which included patient representatives of both POs above mentioned, contributed to the analysis and interpretation of findings, ensuring accurate representation of patient perspectives. Presidents and vice-presidents of Gruppo LES Italiano and APMARR are listed among the authors of this paper due to their invaluable contributions. The results of this analysis are and will be shared with the POs which will then share it with both participants and patients.

## 3. Results

### 3.1. Qualitative Analysis

The qualitative analysis involved 10 participants (9 females and 1 male), contributing to the identification of key topics for further exploration in the quantitative phase. Detailed demographics are provided in Supplementary Table S1.

The participants were predominantly young women: 50% aged 21–40 years, 30% aged 41–50 years, and 20% over 50 years.

The qualitative analysis revealed that patients are not comprehensively managed for all chronic aspects of their disease, and the psychological impact of SLE is often underestimated. Findings indicate that this chronic condition negatively impacts all aspects of patients’ lives: biologically (physical and mental symptoms), personally (psychological distress, frustration, and guilt), and socially (interpersonal relationships, family, and work).

The findings align with those reported in the quantitative section.

### 3.2. Quantitative Analysis

#### 3.2.1. Demographics

A total of 151 Italian patients with SLE participated in the patient listening project (5% male, 95% female). Geographically, 40% were from the North, 32% from the Center, and 28% from the South. The mean age was 43 years, ranging from 18 to 70 years.

**Figure 2:**
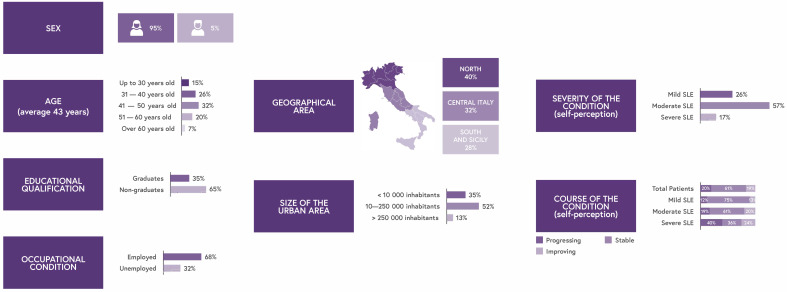
Demographic data.

Detailed demographics, including social factors, education, and employment status, are presented in Figure 2. Patients self-reported their disease severity, stratified into mild (26%), moderate (57%), and severe (17%) SLE categories. The average disease duration was 15.7 years.

#### 3.2.2. 12-Item Medical Outcome Short Form (SF-12)

Comparison of the SF-12 form and a well-known national socio-demographic report (Istat BES Report 2014) revealed that both the Physical Component Summary (PCS) and Mental Component Summary (MCS) scores were significantly lower among patients with SLE compared to the general Italian population, as detailed in Table 1.

Self-reported perception of the disease allowed the stratification of the patients into mild, moderate, and severe SLE.

#### 3.2.3. Descriptive Questions

- Symptom Prevalence and Perceptions

Analysis of patient-reported symptoms identified dizziness and fatigue as the most impactful symptoms (80%), followed by musculoskeletal pain (64%) and joint pain (56%). Additionally, physical pain, confusion, and cognitive deficits (concentration and memory) were reported by 31% of patients. Dermatological and esthetic manifestations were also noted by 33% of patients (Figure S1).

Patients expressed significant concern about disease progression (83%), particularly related to renal issues (mild: 55%, moderate: 48%, severe: 60%) and cardiac complications (mild: 45%, moderate: 33%, severe: 32%).

- Impact of SLE on Daily Life, Employment, and Leisure Activities

**Figure 3:**
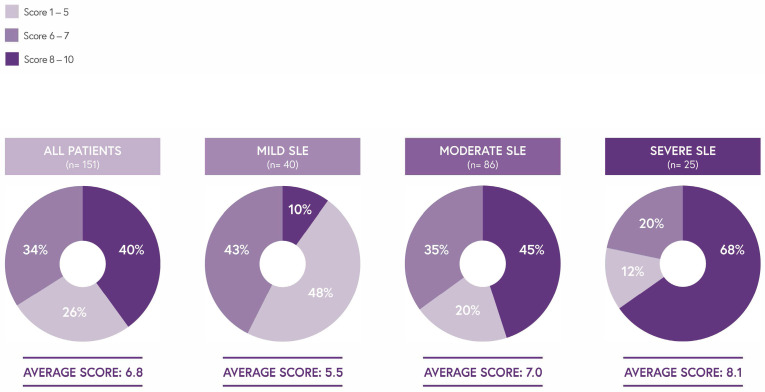
Overall SLE interference with everyday life.

The self-reported impact of the disease on daily life correlated with disease severity (Figure 3). Patients with severe SLE reported higher scores (8.1) compared to those with moderate (7.0) and mild (5.5) SLE on a 1–10 scale (higher scores indicate worse conditions).

This pattern was consistent across various aspects of life, including physical, emotional, and interpersonal interactions (Figure S2A–C).

The impact of SLE on occupational status was also notable (Figure S2D). On average, patients reported missing approximately six weeks of work per year, with 82% needing to take leave or time off due to their condition. Additionally, one-third of SLE patients stated that they had to change jobs because of their diagnosis.

Physical and emotional distress not only disrupted professional life but also affected other daily and leisure activities. Specifically, 76% of participants reported that their physical condition limited the type of work or activities they could engage in, and 82% noted that their emotional health negatively impacted their concentration at work.

- Patient Journey Steps

○ The Diagnosis

This section collected detailed insights into the diagnostic journey of a patient with SLE. General practitioners were identified as the first point of contact for symptoms by 62% of patients, while 37% directly consulted a rheumatologist (multiple responses allowed).

Notably, in 26% of cases, the onset of initial symptoms led to emergency hospitalization (Figure S3A). The most frequently reported early symptoms were limb pain and dizziness, with more severe cases presenting multi-systemic complications at diagnosis (Figure S3B).

The diagnostic process was often prolonged and complex, especially for patients with moderate and severe forms of SLE. Notably, 45% experienced delays longer than two years. On average, it took 2.7 years to receive a diagnosis, typically involving consultations with around five different healthcare professionals (Figure S4).

On average, diagnosis occurred at around 30 years of age. Furthermore, 30% of patients reported that the information provided by healthcare professionals was unclear. Many found clinician explanations overly technical or insufficient, creating uncertainty about treatment plans and disease progression.

Only 20% of respondents had access to psychological support in the early stages of the disease, and 80% indicated that they were not referred to such services. This aligns with literature emphasizing the emotional toll of SLE and reinforces the need for integrated mental health services.

○ Follow-Up

During follow-up, patients with SLE reported undergoing an average of 16–20 medical visits, including consultations with psychologists and nutritionists. Due to long waiting lists in the Italian National Health Service (SSN), patients often resorted to private healthcare, with over one-third of visits conducted privately, resulting in an average annual out-of-pocket cost exceeding €700 (Figure S5).

When asked about multidisciplinary care, only 44% of patients reported being managed by a multidisciplinary team. Additionally, 20% of patients faced relocation or long-distance travel, sometimes to different regions, to access specialized care.

Once connected with a reference center, 32% of patients expressed satisfaction with the care received, while 29% were dissatisfied. Dissatisfaction was more common among patients not treated in specialized SLE centers. Higher satisfaction scores were typically reported in facilities offering structured care pathways and demonstrating coordination among specialists.

○ Therapeutic Regimen

To evaluate patients’ understanding and concerns regarding pharmacological therapy, participants were asked to report their current medications.

**Figure 4:**
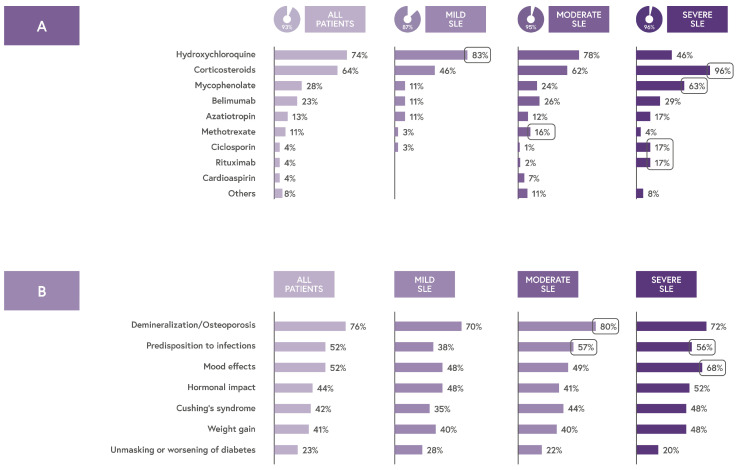
(**A**) Pharmacological treatments in patients with SLE. (**B**) Main concerns related to corticosteroid therapy according to patients with SLE.

The majority (93%) were on pharmacological therapies. Hydroxychloroquine (HCQ) was the most commonly used medication (74%), although it was prescribed less frequently in severe cases (46%). Corticosteroids were the second most common treatment (64%), with use increasing alongside disease severity (46% in mild, 62% in moderate, and 96% in severe cases) (Figure 4A).

Patients with severe SLE also reported using mycophenolate (63%), cyclosporine (17%), and belimumab (29%).

Although the latest EULAR recommendations advise minimizing and, when possible, avoiding corticosteroid use due to long-term adverse effects, this medication class remains widely prescribed [13].

Patients expressed significant concern over corticosteroid side effects, particularly the risk of bone demineralization. Additional concerns, such as infection risk and mood changes, became more prominent as disease severity increased (Figure 4B). Concerns about potential adverse effects such as weight gain and diabetes were reported less frequently.

Adherence to treatment was suboptimal, with only 65% of patients reporting full compliance with medical prescriptions. Among patients with mild SLE, 26% admitted to occasionally forgetting to take their medications (Figure S6). As anticipated, most patients expressed a preference for home-based treatments (68%).

○ Critical Issues and Unmet Needs

Patients were asked to identify the main challenges they face in managing SLE and to highlight any unmet needs.

The most frequently reported concern was the adverse effects of pharmacological treatments (66%), followed by the financial burden of purchasing medications, supplements, and sunscreen products (62%). Approximately 50% expressed worries about the cost of private medical visits and the long waiting lists in public healthcare services.

Consequently, key unmet needs included the following:

- Increased financial support to cover therapy-related expenses (61%);
- A more multidisciplinary approach to treatment, particularly to manage therapy-related adverse events (59%);
- Establishment of more specialized SLE centers across Italy to improve access to care (56%) (Figure S7).

Additionally, patients emphasized the need for the following:

- Greater economic assistance for purchasing health-related consumer goods (29%);
- Stronger interdisciplinary coordination among healthcare providers (31%);
- Easier access to benefits under Law 104 to facilitate medical visits and examinations (29%).

### 3.3. The Advisory Board Phase

The Advisory Board facilitated collaborative discussion between clinicians and patient representatives, providing valuable advice, insights, and recommendations.

Several key issues emerged during this phase:

Awareness of Disease Progression and Treatment Risks: Patients tended to focus on current symptoms, underestimating long-term risks of organ damage and adverse effects from both disease and treatment. Clinicians emphasized the seriousness of complications, such as renal damage and therapy-related diabetes.

Therapy Adherence: Although patients acknowledged the importance of adherence, clinicians highlighted that adherence rates below 70% remain suboptimal.

Perception of Corticosteroid Use: Patients viewed corticosteroids as essential for disease control, despite concerns over side effects. Clinicians and EULAR guidelines advocate reducing or avoiding corticosteroid use when possible.

Role of Non-Pharmacological Treatments: Patients often underestimated the benefits of exercise, nutrition, and lifestyle changes, while clinicians stressed their importance.

Multidisciplinary Care Approach: Patients expressed a desire for consistent care from a multidisciplinary team, valuing continuous contact and psychological support. Clinicians noted that involving multiple specialists at different stages could optimize treatment strategies.

## 4. Discussion

This study aimed to provide a comprehensive overview of unmet needs and quality-of-life (QoL) challenges among Italian patients with systemic lupus erythematosus (SLE), based on findings from the PaLESiamoci! project and the Advisory Board (AB) discussions. The results highlight several critical issues that stakeholders, including institutions, clinicians, patient organizations (POs), and caregivers, should address to improve patient outcomes. Consistent with previous reports, the collected data confirm that SLE profoundly affects all dimensions of patients’ lives, impacting physical health, psychological well-being, and social interactions, which together shape overall QoL.

The present findings align with a growing body of literature emphasizing the importance of patients’ perspectives in understanding the burden of SLE. In particular, Petrocchi et al. recently summarized 26 studies exploring patients’ lived experiences, reinforcing the relevance of capturing psychosocial and daily-life dimensions of the disease [14]. The current study contributes to this literature by focusing specifically on the Italian population and by integrating both qualitative and quantitative data with expert discussions, offering a broader and more contextually relevant perspective.

A major strength of this analysis lies in its dual focus: it quantifies the burden of SLE and contextualizes the findings through expert insights. The SF-12 results revealed that patients scored substantially lower on both the Physical Component Summary (PCS, mean 37.8; −26.1% vs. the general population) and the Mental Component Summary (MCS, mean 35.0; −29% vs. the general population). These findings are consistent with the qualitative results, confirming that both the disease itself and its pharmacological management have a strong influence on patients’ daily and professional lives.

Regarding pharmacological therapy, the widespread use of glucocorticoids, often at increasing doses with disease severity, remains a major concern. AB discussions revealed that many patients rely heavily on corticosteroids to manage symptoms and disease progression while underestimating their long-term adverse effects. Interestingly, concerns about metabolic complications such as weight gain or diabetes appeared lower among patients than among clinicians, suggesting possible gaps in risk communication and shared decision-making.

Beyond clinical management, the data highlight considerable bureaucratic and logistic barriers. The prolonged diagnostic journey, with an average delay of nearly three years, points to the need for greater disease awareness among general practitioners and non-specialist physicians to promote earlier detection. Patients also emphasized the scarcity of specialized SLE centers nationwide and the lack of structured care pathways ensuring continuity, coordination, and psychological support. These findings suggest that healthcare policies should prioritize equitable access to multidisciplinary care and psychological services [15,16,17,18].

A particularly noteworthy point from the AB concerned differing interpretations of what constitutes a multidisciplinary approach. For patients, it often means being visited by multiple specialists during consultations, while clinicians described multidisciplinary care as collaboration between specialists through dedicated case discussions. Despite these divergent views, both groups acknowledged the essential role of integrated, coordinated care in optimizing treatment outcomes and patient satisfaction.

Furthermore, patients appeared to underestimate the value of non-pharmacological strategies such as exercise, lifestyle adjustments, and nutrition in disease management. This perception may reflect suboptimal communication between patients and healthcare professionals, emphasizing the need for clearer education on comprehensive care approaches.

Overall, the insights emerging from this project have direct implications for healthcare planning and policy development. Since the results reflect the real-world impact of SLE on the Italian healthcare system, they could inform future clinical guidelines, strengthen multidisciplinary collaboration, and shape educational initiatives aimed at both patients and healthcare providers [19]. Future research should expand these findings by including longitudinal assessments to evaluate how targeted interventions addressing these unmet needs could improve QoL and disease outcomes over time.

## 5. Conclusions

This analysis, through the “PaLESiamoci!” patient listening project and subsequent Advisory Board, provided essential, patient-derived insights into the quality of life and unmet needs of Italian patients with SLE. The findings confirm that psychological distress, fatigue, and the long-term management of side effects, particularly corticosteroid use, are major challenges that require immediate attention. The project highlighted the urgent need for a more structured, multidisciplinary approach to care, improved patient education regarding long-term risks and non-pharmacological treatments, and better access to specialized centers and financial support. These outcomes are a crucial resource for developing patient-centered strategies aimed at improving clinical practice and optimizing care pathways for SLE patients in Italy.

## Figures and Tables

**Table 1:** SF-12 data. Absolute value scores resulted from the 12-item short form. Physical Component Summary (PCS) and Mental Component Summary (MCS) scores were compared to reference scores. * Istat BES Report 2014 [12].

	PCS-12 Score	Δ(PCS-PCSr)	MCS-12 Scores	Δ(MCS-MCSr)
Italian reference *	**51.2**	**0**	**49.0**	**0**
Mild SLE (n = 40)	43.3	−7.9	38.4	− 10.6
Moderate SLE (n = 86)	36.9	− 14.3	34.7	14.3
Severe SLE (n = 25)	32.0	− 19.2	30.3	− 18.7
**Average SLE (n = 151)**	**37.8**	− **13.4**	**35.0**	− **14.0**

## Data Availability

The data that supports the findings of this study is available upon reasonable request to the corresponding author. The data is not publicly available due to privacy and ethical restrictions.

## Supplementary Materials

The following supporting information can be downloaded at https://www.mdpi.com/article/10.3390/jcm14238498/s1. Figure S1: Symptoms perception in SLE patients; Table S1: Qualitative analysis: sample demographics. Number of patients is reported, sorted by age both at the time of investigation and at the time of diagnosis; Figure S2: A. Impact of SLE on physical activity, B. Impact of SLE on psychological wellbeing, C. Impact of SLE on interpersonal and social interactions, D. Impact of SLE on Jobs and other daily activities; Figure S3: A: HCP consulted, B: first recognized symptoms; Figure S4: Time for the diagnosis and average number HCP consulted before being diagnosed; Figure S5: Follow-up visits and management of the patients by SLE centers; Figure S6: Therapeutic adherence in SLE patients; Figure S7: Critical issues and unmet needs for SLE Italian patients.

## Abbreviations

The following abbreviations are used in this manuscript:

**Abbreviation**	**Meaning**
AB	Advisory Board
AE	Adverse Event
APMARR	Associazione Nazionale Persone con Malattie Reumatologiche e Rare
CAWI	Computer-Assisted Web Interviewing
EULAR	European Alliance of Associations for Rheumatology
GSK	GlaxoSmithKline
IQVIA	Name of the contracted survey service provider
Istat	Istituto Nazionale di Statistica
MCS	Mental Component Summary
PCS	Physical Component Summary
PO	Patient Organization
PPI	Patient and Public Involvement
PROM	Patient-Reported Outcome Measure
QoL	Quality of Life
SF-12	12-item Medical Outcome Short Form
SLE	Systemic Lupus Erythematosus
SSN	Servizio Sanitario Nazionale (Italian National Health Service)

## Appendix A

### Appendix A.1

#### Appendix A.1.1. Patient Diary: “Diario Pazienti LES”

**Question ID**	**Question**	**Response Type/Options**
H1	Personal background (age, education, lifestyle, personality, etc.)	Open-ended (written text)
H2	Description of how diagnosis was communicated	Open-ended (written text, optional image)
H3	First thoughts and emotional reaction to diagnosis	Open-ended (written text, optional image)
H4	Perception of SLE (metaphors, symbolic description)	Open-ended (written text)
H5	Imagined dialogue with SLE	Open-ended (dialogue bubbles)
H6	Experience and emotions related to therapy	Open-ended (written text, optional image)
H7	Relationship with the physician(s)	Open-ended (written text, analogies/examples)
H8	Challenges in everyday life and moments of greater difficulty	Open-ended (written text, optional image)
H9	Emotional and relational impact (identity, body image, relationships)	Open-ended (written text)
H10	Unmet needs, suggestions for services, and what would help the most today	Open-ended (written text)

#### Appendix A.1.2. Interview Discussion Guide: “Questionario PALESIAMOCI”

**Question ID**	**Question**	**Response Type/Options**
I1	Patient’s illness journey: symptoms, diagnosis, emotional reaction	Semi-structured interview
I2	Communication of diagnosis and understanding of disease	Semi-structured interview
I3	Pathway to and experience with treatment center	Semi-structured interview
I4	Relationship with medical specialist(s) and experience during visits	Semi-structured interview
I5	Current and past therapies, treatment satisfaction	Semi-structured interview
I6	Emotional relationship with therapy (ally, burden, etc.)	Semi-structured interview
I7	Difficulties in managing SLE in daily life	Semi-structured interview
I8	Emotional impact, identity changes, self-perception	Semi-structured interview
I9	Effect on work, relationships, and support from caregivers	Semi-structured interview
I10	Experience with follow-up and multidisciplinary care	Semi-structured interview
I11	Use of informational sources and channels	Semi-structured interview
I12	Role and experience with patient associations	Semi-structured interview
I13	Suggestions for improving the patient’s care journey and support system	Semi-structured interview

### Appendix A.2

#### Appendix A.2.1. English Translation of the SLE Patient Questionnaire

##### Section A—Demographics & Clinical Information

**Question ID**	**Question**	**Response Type/Options**
A1	What is your gender?	Male/Female
A2	What is your age?	Numeric input
A3a	Which region do you live in?	Single choice
A3b	Which province do you live in?	Single choice
A3c	Approximate number of inhabitants in your city?	Numeric input
A4	What is your highest level of education?	None/Primary/Middle/High school/University/Postgraduate
A5	Age at which you were diagnosed with SLE?	Numeric input
A6	Time between first symptoms and diagnosis?	Weeks/Months/Years + Numeric input/I don’t remember
A7	Which doctors did you consult before diagnosis?	Multiple choice
A8	Number of doctors seen before SLE diagnosis?	Numeric input
A9	Which doctor diagnosed your SLE?	Multiple choice
A10	Symptoms that led you to seek medical help?	Multiple choice
A11	Current symptoms?	Multiple choice
A12	Symptoms that most affect your daily life?	Multiple choice
A13	Symptoms that worry you the most?	Multiple choice
A14	How would you describe your disease?	Mild/Moderate/Severe
A15	How is your disease currently evolving?	Stable/Improving/Progressing
A16	Was the diagnosis clearly explained by your doctor?	Very/Quite/A little/Not at all
A17	Did you receive advice from healthcare professionals?	Yes, very/Yes, somewhat/Yes, but not much/No advice received
A18	Did you receive support (psychologist, nurse, etc.)?	Yes, very useful/Yes, somewhat/Yes, not useful/No support

##### Section B—Follow-Up & Healthcare Use

**Question ID**	**Question**	**Response Type/Options**
B1	Are you followed by a multidisciplinary SLE center?	Yes/No
B2	Which specialists are part of the team?	Multiple choice
B3	Which specialists have you seen in the past year?	Multiple choice
B4	Number of visits per specialist in the past year?	Numeric input
B5	Public vs. private visits?	Numeric input
B6	Who is your primary specialist?	Single choice
B7a	Are all specialists in the same structure?	Yes/No
B7b	Is the center in your city/region?	Same city/Same region/Different region
B8	Why did you go private for some visits?	Multiple choice
B9	Annual cost of private visits?	Numeric input (Euro)
B10	Why did you choose your treatment center?	Multiple choice
B11	Satisfaction with center (various aspects)?	Scale 1–10
B12	Overall satisfaction with center?	Scale 1–10
B13	How many centers have you changed?	Numeric input
B14	Can you complete all prescribed exams/visits?	Always/Sometimes/Often not/Other
B15	Is your center a recognized reference for SLE?	Absolutely/Quite/Not really/No

##### Section C—Treatments & Adherence

**Question ID**	**Question**	**Response Type/Options**
C1	Is your SLE in remission or active phase?	Remission/Active
C2	Are you currently on medication for SLE?	Yes/No
C3a–d	What drugs do you take, and how?	Name, route, dosage, place
C4a	Satisfaction with administration mode?	Very/Somewhat/Not much/Not at all
C4b	Preferred administration mode?	Open-ended
C4c	Preference: home vs. hospital?	Visual analog scale
C5	Can you follow your daily therapy?	Multiple choice
C6	Did you purchase non-reimbursed products?	Yes/No by item
C7	Annual cost of non-reimbursed products?	Numeric input (Euro)
C8	What corticosteroid effects worry you most?	Multiple choice

##### Section D—Daily Life Impact

**Question ID**	**Question**	**Response Type/Options**
D1	How would you rate your general health?	Excellent to Poor scale
D2	Limitations in moderate physical activities?	Yes, a lot/A little/No
D3	Limitations climbing stairs?	Yes, a lot/A little/No
D4	Physical health affected work/daily activities?	Yes/No—Reduced output/Limited tasks
D5	Emotional state affected work/daily activities?	Yes/No—Reduced output/Lost focus
D6	Pain interference with usual work?	Scale 1–5
D7	How often felt calm/energetic/sad?	Scale: Always to Never
D8	Interference with social/family/friend life?	Scale: Always to Never
D9	Overall interference with daily life?	Scale 1 (Very little) to 10 (Very much)
D10	Agreement with SLE impact statements?	Scale 1 (Not at all) to 10 (Completely agree)

##### Section E—Work & Insurance

**Question ID**	**Question**	**Response Type/Options**
E1	What is your current work status?	Employed/Unemployed/Retired/Student/Other
E2	How many work days lost per year?	Numeric input
E3	Did you change job due to SLE?	Yes/No
E4	Are you hired as a protected category?	Yes/No
E5	Do you use Law 104 benefits?	Yes/No
E6	Do you take time off for SLE visits?	Yes/No
E7–E10	Job/study changes due to SLE?	Yes/No + Reasoning
E11	Job reduction due to SLE?	Yes/No + How
E12–E13	Student-specific questions?	Days missed/Course choice influence
E14	Do you receive disability or care benefits?	Multiple choice
E15	Do you have private/enterprise insurance?	Yes/No
E16	Was insurance denied due to SLE?	Yes/No

##### Section F—Critical Issues & Patient Needs

**Question ID**	**Question**	**Response Type/Options**
F1	Most critical issues managing SLE?	Multiple choice
F2	Top 3 critical issues?	Ranking
F3	Helpful interventions for SLE patients?	Multiple choice
F4	Top 3 helpful supports?	Ranking
F5	Other information sources used?	Multiple choice

### Appendix A.3

Topics and questions asked during AB:

1. **Adherence:** In your opinion, are patients aware of the importance of continuous therapeutic adherence in SLE, including in remission or relapse moments?
2. **Awareness:** What are the drugs that patients perceive as essential to stop the progression of the disease? Does this information derive from clinicians or other sources?
3. **Awareness:** Are patients aware of non-pharmacological strategies to reduce the progression of the disease?
4. **Awareness:** Considering SLE-related organ damage progression, is there awareness among patients on how irreversible this could be?
  Are they aware that organ damage could be correlated either to disease progression or to therapy?
  How do patients inform (through the clinician, PAG, autonomously)?
  What can be done to improve awareness?
5. **Patients’ journey:** How is patients’ journey? Do they have discontinuity periods, considering cure reference points? Does this impact adherence?
6. **Implementation:** Which activities could the industry implement to understand and meet SLE patients’ need for the best?
