# Supplementary material for: Italian Systemic Lupus Erythematosus (SLE) Patients: Overview of Their Quality of Life and Unmet Needs
Source: J Clin Med. 2025 Nov 30;14(23):8498. doi: 10.3390/jcm14238498 (PMC12693608; doi:10.3390/jcm14238498)
Supplement: Supplementary file 1 [file jcm-14-08498-s001.zip › jcm-4008609-supplementary.pdf]

Journal of
Clinical Medicine

MDPI

Supplementary Materials: Supplementary information Table S1.

Disease duration (years)	Age of Participants (years)			
	21-40	41-50	51-60	>60
≤5		1		
6-10	1	1		
15-20	3	1		
>20	1		1	1

Table S1. Qualitative analysis: sample demographics. Number of patients is reported, sorted by age both at the time of investigation and at the time of diagnosis.

Supplementary information Figure S1

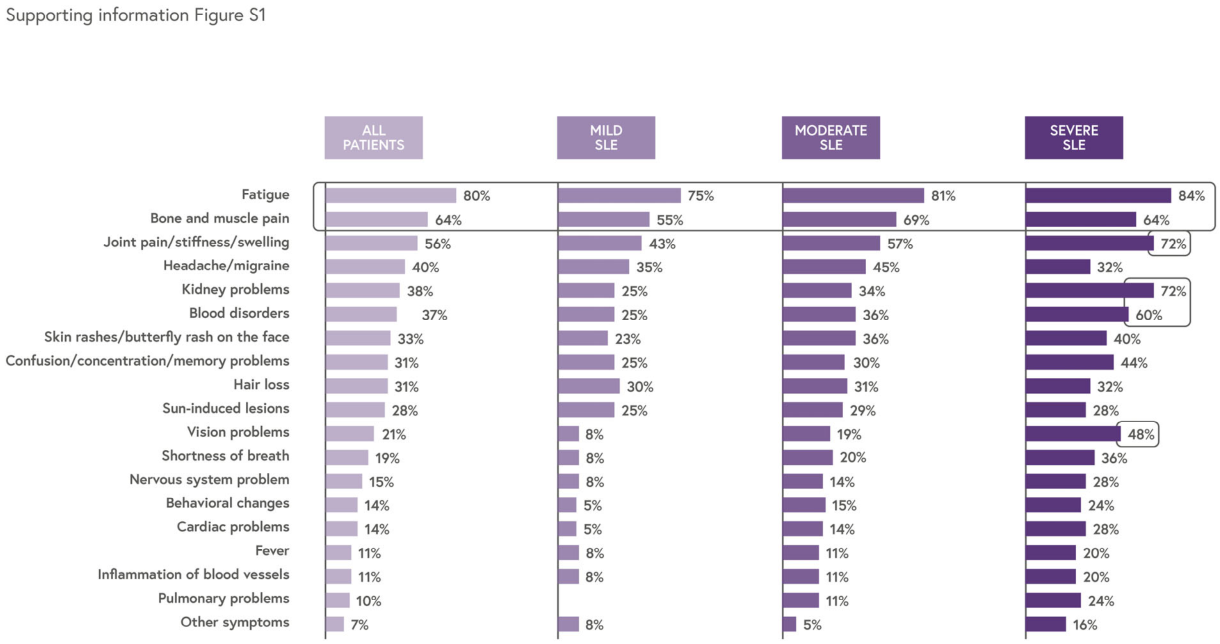

Figure S1: symptoms perception in SLE patients

Supplementary information Figure S2

A. Impact of SLE on physical activity

Supporting information Figure S2

A. Impact of SLE on physical activity

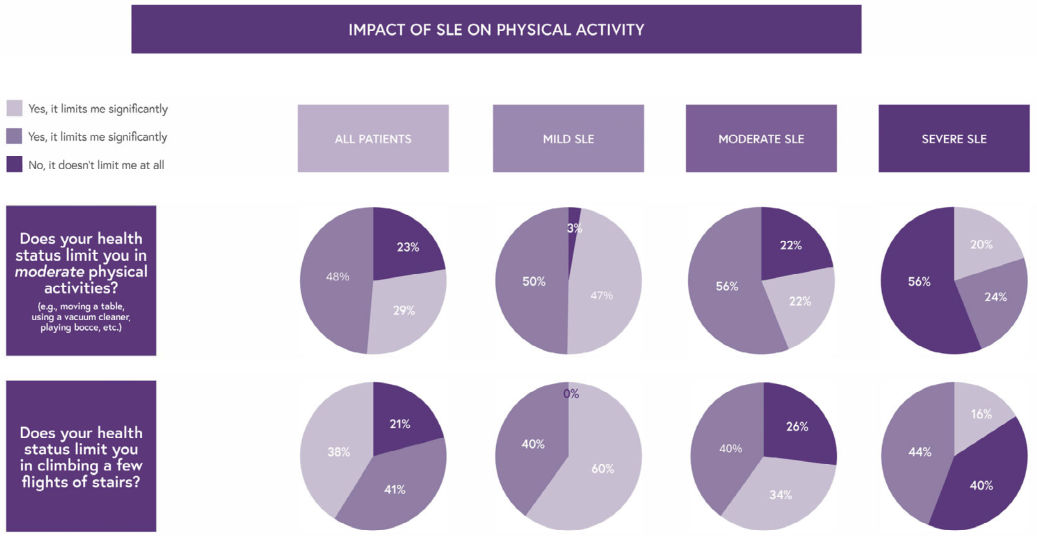

B. Impact of SLE on psychological wellbeing

Supporting information Figure S2

B. Impact of SLE on psychological wellbeing

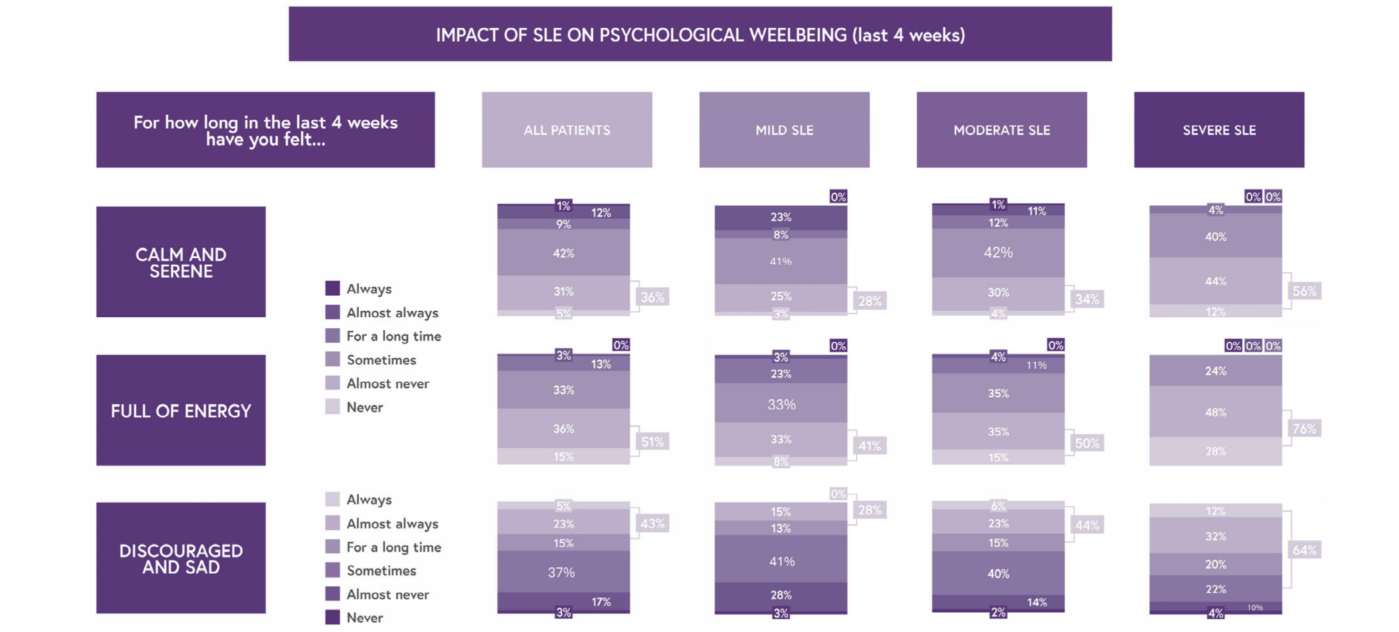

C. Impact of SLE on interpersonal and social interactions

Supporting information Figure S2

C. Impact of SLE on interpersonal and social interactions

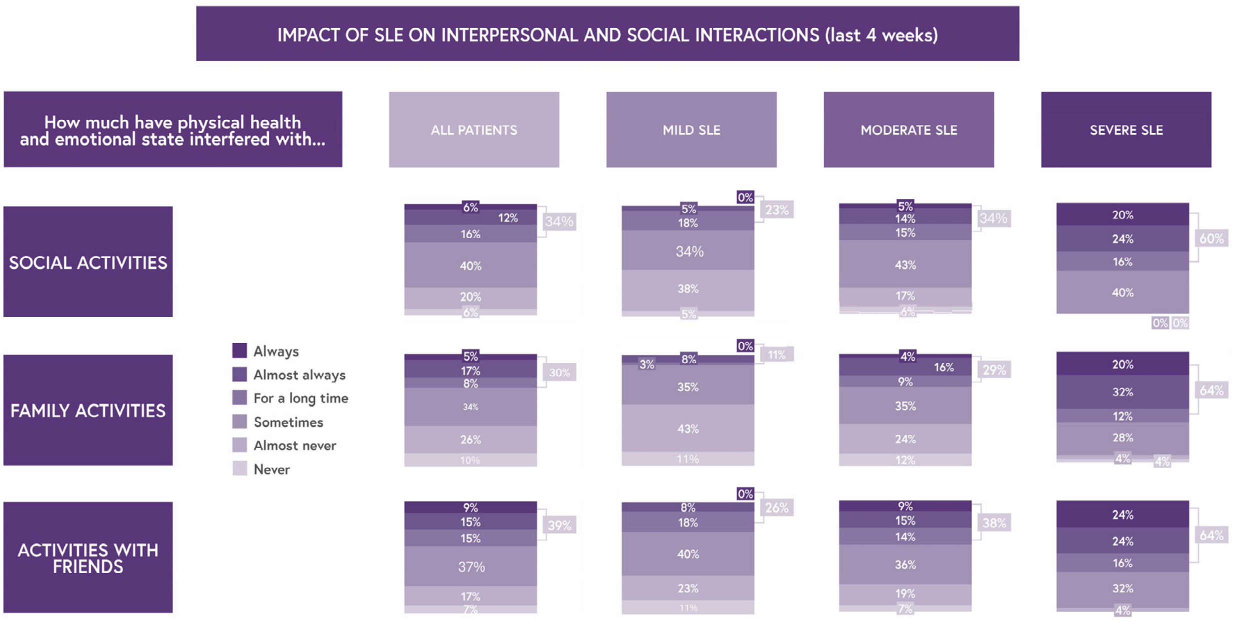

D. Impact of SLE on Jobs and other daily activities

Supporting information Figure S2

D. Impact of SLE on Jobs and other daily activities

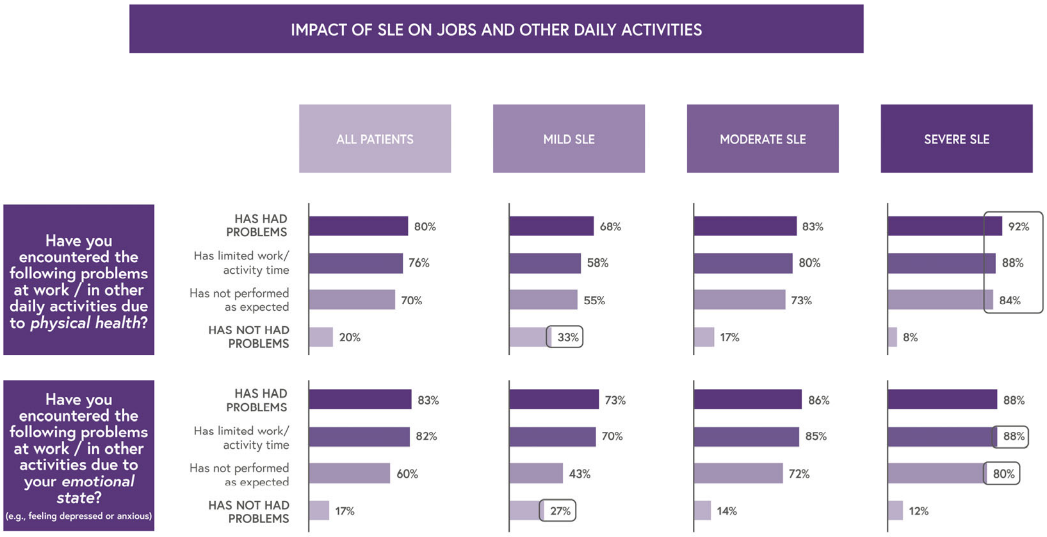

Supplementary information Figure S3

Supporting information Figure S3

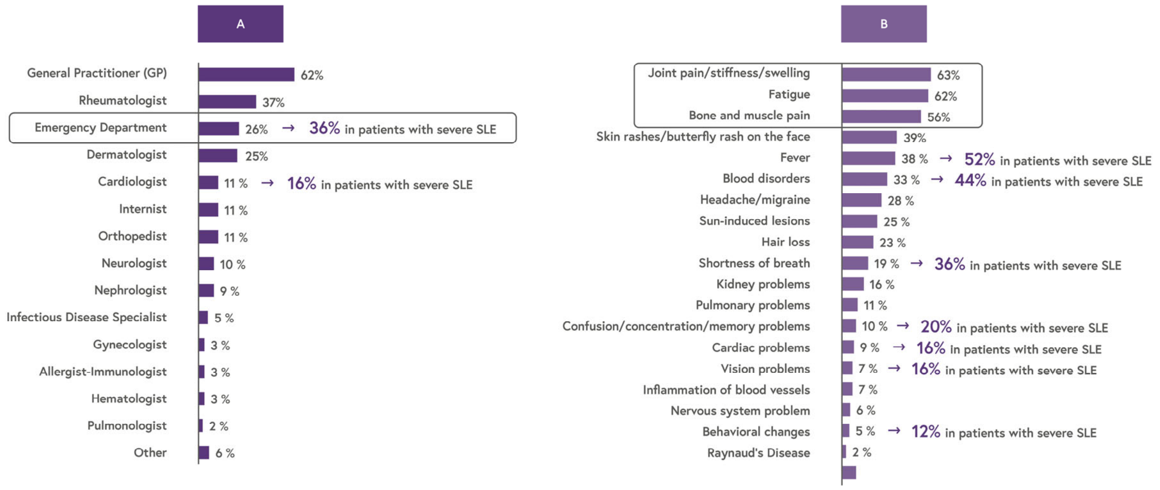

Figure S3: A: HCP consulted, B: first recognized symptoms

Supplementary information Figure S4

Supporting information Figure S4

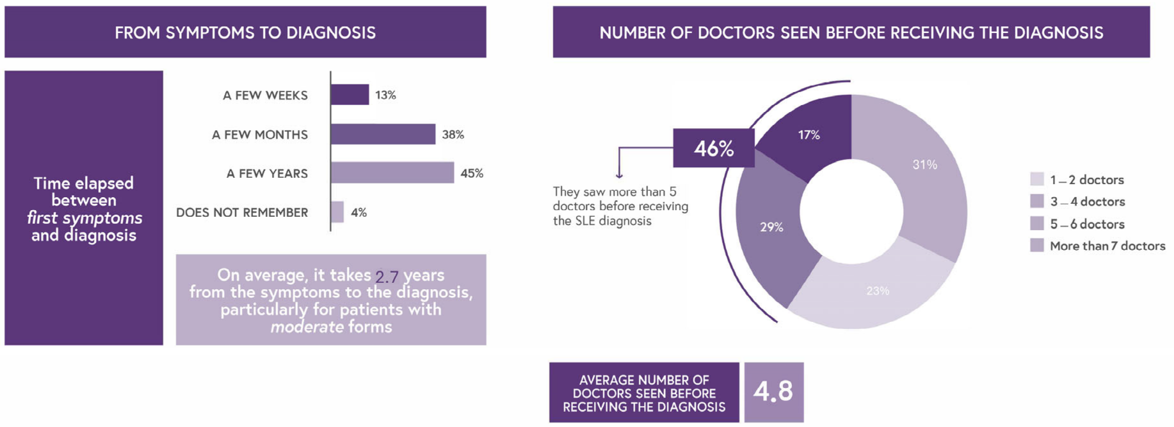

Figure S4: Time for the diagnosis and average number HCP consulted before being diagnosed

Supplementary information Figure S5

Supporting information Figure S5

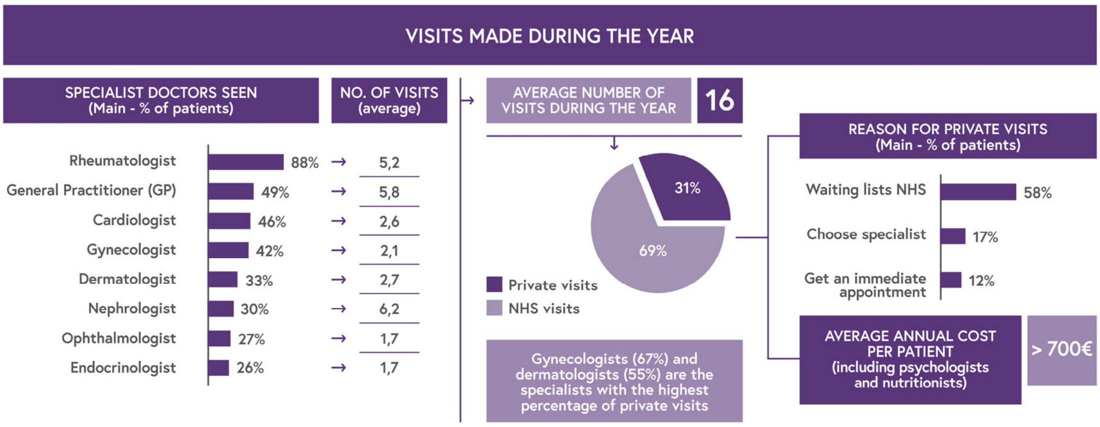

Figure S5: Follow-up visits and management of the patients by SLE centers.

Supplementary information Figure S6

Supporting information Figure S6

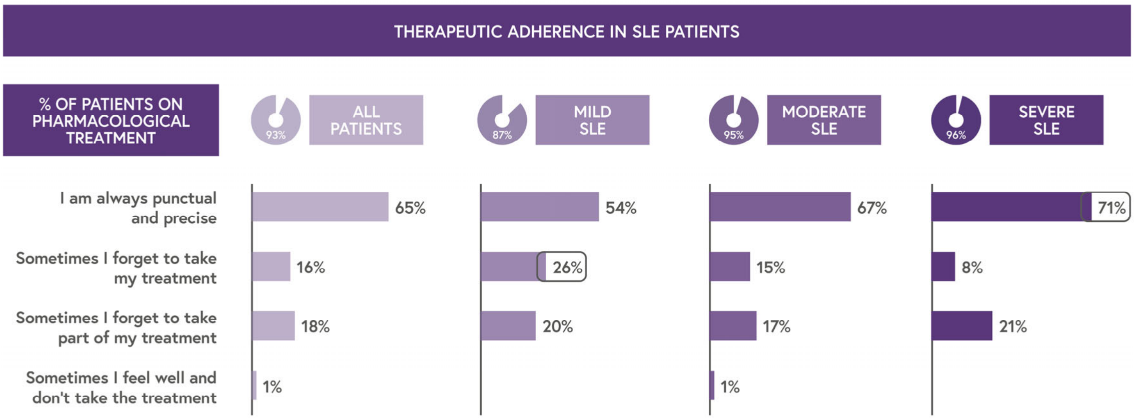

Figure S6: Therapeutic adherence in SLE patients.

Supplementary information Figure S7

Supporting information Figure S7

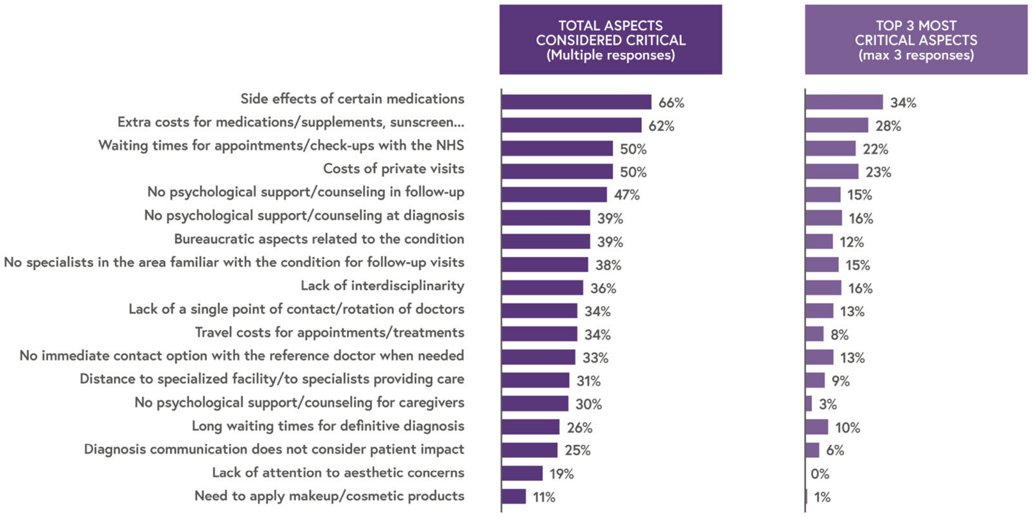

Supporting information Figure S7

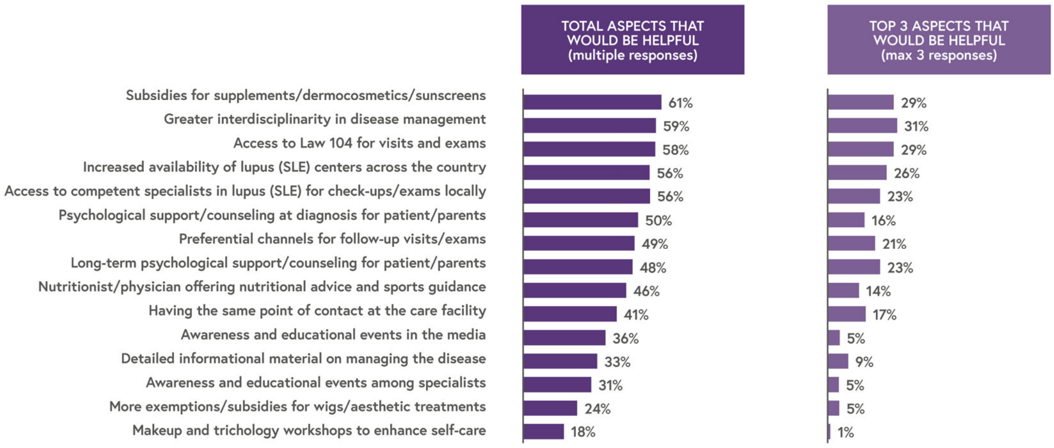

Figure S7: Critical issues and unmet needs for SLE Italian patients.
